# Prediction of Weight and Body Condition Score of Dairy Goats Using Random Forest Algorithm and Digital Imaging Data

**DOI:** 10.3390/ani15101449

**Published:** 2025-05-16

**Authors:** Mateus Alves Gonçalves, Maria Samires Martins Castro, Eula Regina Carrara, Camila Raineri, Luciana Navajas Rennó, Erica Beatriz Schultz

**Affiliations:** 1 Department of Animal Science, Federal University of Viçosa, University Campus, PH. Rolfs Ave, Viçosa 36570-900, MG, Brazil; mateus.a.goncalves@ufv.br (M.A.G.); maria.s.castro@ufv.br (M.S.M.C.); eulacarrara@gmail.com (E.R.C.); lucianarenno@ufv.br (L.N.R.); 2 School of Veterinary Medicine and Animal Science, Federal University of Uberlândia, Uberlândia 38408-144, MG, Brazil; camilaraineri@ufu.br

**Keywords:** artificial intelligence, precision livestock farming, small ruminants

## Abstract

The aim of study was to evaluate the use of digital images to predict body weight (BW) and classify the body condition score (BCS) of dairy goats. A total of 154 female Saanen and Alpine goats were used to obtain eight body measurements features from digital images: withers height (WH), rump height (RH), body length (BL), chest depth (D), paw height (PH), chest width (CW), rump width (RW), rump length (RL). All animals were weighed using manual scales, and their BCS was evaluated on a scale of 1 to 5. For classification purposes, the BCS was grouped into three categories: low (1–2), moderate (2–3), and high (>3). Pearson’s correlation analysis and the Random Forest algorithm were performed. It was possible to predict BW using image features with an R^2^ of 0.87, with D (22.14%), CW (18.93%) and BL (15.47%) being the most important variables. For the BCS, the classification accuracy was 0.4054 with the CW (20.38%) the most important variable followed by RH and RL with 15.78% and 12.63%, respectively. It was concluded that digital image features can be used to obtain precise estimates of body weight, but it is necessary to increase data variability to improve the BCS classification of dairy goats.

## Simple Summary

On dairy goat farms, monitoring weight and body condition is essential for proper animal management. Currently, these measurements are recorded manually, which can be time-consuming and inconsistent. This study explores the use of digital images and artificial intelligence to estimate weight and body condition in dairy goats. By analyzing images of 154 dairy goats, we tested whether machine learning models could accurately predict these traits. The results showed that digital imaging is a reliable method for estimating body weight, providing a faster and more cost-effective alternative to traditional techniques. However, the approach was less effective in classifying body condition, suggesting that further improvements are needed. This research highlights the potential of digital tools to enhance farm efficiency, reducing labor while improving data accuracy for better decision-making in animal care.

## 1. Introduction

Measuring the weight and body condition score (BCS) of dairy goats is essential for monitoring their nutritional and health status, which significantly impacts the productive and economic farm efficiency [1,2]. Although important, continuous body weight (BW) monitoring in dairy systems as highlighted by Martins et al. [3] is labor-intensive, requiring specialized equipment, manual labor, and animal restraint. Furthermore, body condition scoring depends on trained assessors and is subject to variability and subjectivity between evaluations. Advances in technology are enabling the assessment of body weight and condition score through imaging techniques, making the process faster, more accurate, and non-invasive [4].

Li and Thang [5] highlighted that digital image, combined with artificial intelligence tools such as deep learning, can be used to obtain morphometric data from cattle and goats. Zhang et al. [6] demonstrated a strong correlation between body size measurements from images and body weight in sheep, with body length being the most significant predictor. Additionally, Iqbal et al. [7] found that machine learning methods, specifically the Random Forest and Gradient Boosting Machine algorithms, provided higher accuracy with versatility in predicting body weight from goat body measurements.

However, research on developing technologies to predict the BW and BCS of dairy goats remains limited, highlighting the need for further studies to enhance the effectiveness of digital imaging applications. Therefore, this study aimed to evaluate the use of digital images and the random forest algorithm to predict BW and classify BCS in female dairy goats.

## 2. Materials and Methods

The study was conducted at the Federal University of Viçosa (UFV), located in the municipality of Viçosa, Minas Gerais, Brazil. The project was approved by the Ethics Committee for the Use of Production Animals (CEUAP; protocol 60/2023).

A total of 154 female goats of the Saanen (*n* = 77) and Alpine (*n* = 77) breeds were used, distributed across the following age categories: pre-weaning (*n* = 30, age 1–3 months), post-weaning (*n* = 24, age 4–14 months) and lactating/non-pregnant (primiparous and multiparous) (*n* = 100, age > 15 months). The animals were in an intensive system in collective free-stall stalls with access to the solarium (except for the pre-weaning animals, who were kept in suspended cages) and were equipped with a feeding trough, drinking trough (water ad libitum), salt trough, and sawdust floor covering.

**Figure 1:**
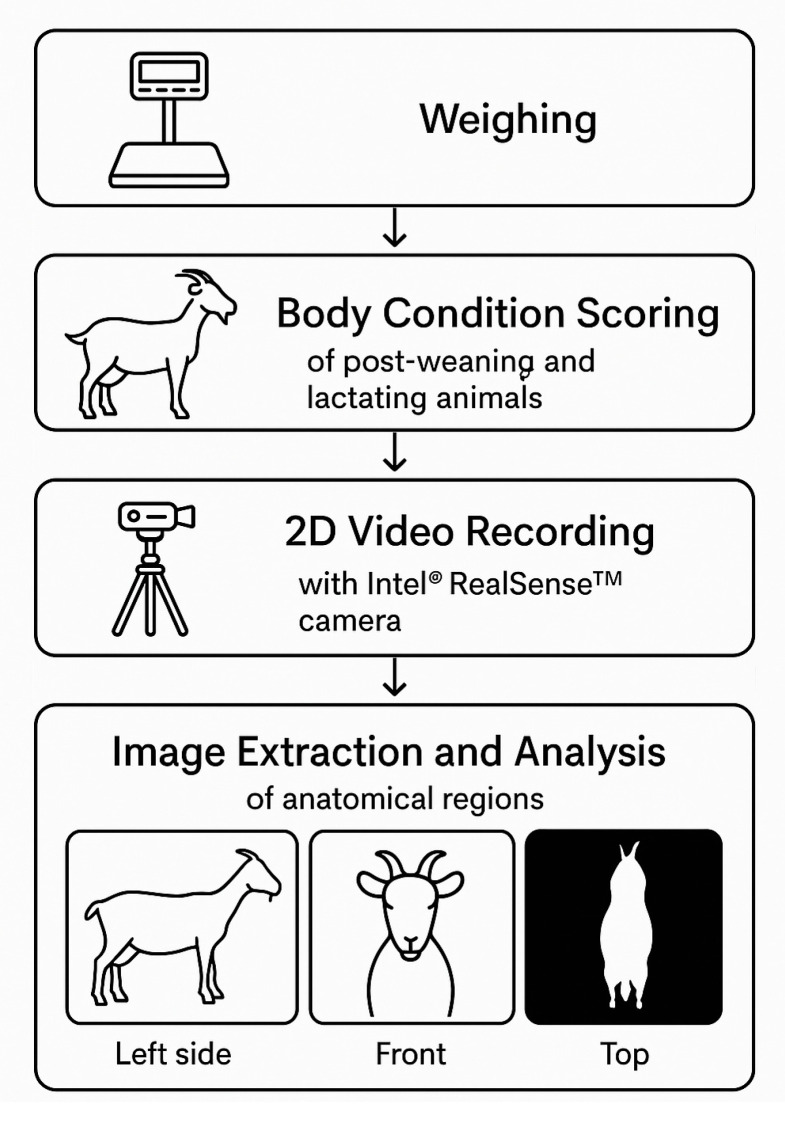
Methodological framework for measuring and collecting images of dairy goats.

For traditional measurements, all animals were weighed once using a fixed digital scale Toledo 2099 (São Bernardo do Campo, Brazil). For body condition score (BCS), only animals in the post-weaning and lactation phases (*n* = 124) were evaluated once by three trained evaluators through palpation of the lumbar region, paying specific attention to the spine, including the spinous and transverse processes using a five-point scale (1 to 5) with 0.5-point increments [8]. Simultaneously, one-minute two-dimensional (2D) video recordings of each animal were captured under natural lighting conditions using an Intel^®^ RealSense™ D435 camera (Santa Clara, CA, USA) mounted on a professional tripod. Individual recordings were taken of three anatomical regions: the left side, front (sternum), and upper (rump). The camera was positioned at a fixed distance of 1.80 m from the animal and set at two heights from the ground: 67 cm for capturing the left side and front views, and 135 cm for the upper view (Figure 1).

**Figure 2:**
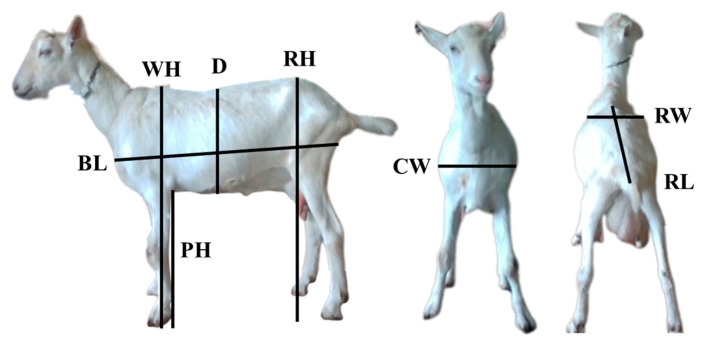
Morphometrics features for dairy goats. WH = withers height; RH = rump height; RW = rump width; RL = rump length; BL = body length; D = chest depth; CW = chest width; PH = paw height.

After image collections, a single frame from each anatomical region was extracted using Intel Viewer software v2.55.1 version. The images, with a resolution of 640 × 480 pixels, were saved in PNG format and processed in ImageJ software 1.54g version. Measuring points were used to obtain morphometric features in pixels: withers height (WH), measured from the highest point of the scapula to the ground; rump height (RH), from the highest point of the rump to the ground; body length (BL), from the anterior shoulder to the point of tail insertion; chest depth (D), from the dorsal line to the deepest point of the thorax; paw height (PH), from the highest point of the front paw to the ground; chest width (CW), from the widest point of the chest to its corresponding opposite point; rump width (RW), at the widest point of the rump; and rump length (RL), from the most anterior to the most posterior point of the rump (Figure 2) [9]. Reference points from the environment were used in the recordings to convert pixel measurements into real-world values (cm).

A descriptive analysis was conducted to summarize the data. Pearson’s correlation was performed at a 5% significance level to assess the relationship between morphometric image features and body weight (BW). Correlations were classified as low (0.00–0.29), moderate (0.30–0.59), high (0.60–0.89), very high (0.90–0.99), and perfect (1.00) [10].

For BW prediction and BCS classification, the dataset was split into 70% for training and 30% for testing, considering the pre-weaning, post-weaning, and lactating/non-pregnant categories for BW, and low (1–2), moderate (2–3), and high (>3) categories for BCS, respectively.

Using morphometric image features, the random forest (RF) machine learning algorithm was optimized by grid search minimizing the root-mean-square error (RMSE) through cross-validation (5-folds) with training data. The importance of the variables calculated in the final model after optimization showed as percentage. The model precision and accuracy for body weight (BW) prediction with test dataset was evaluated using the coefficient of determination (R^2^), RMSE, and mean absolute error (MAE). For body condition score (BCS) prediction, model performance was evaluated using test data with the confusion matrix.

## 3. Results

### 3.1. Prediction of Body Weight

The correlations between body weight (BW) and the morphometric features obtained via imaging were significant (*p* < 0.05) and positive (Table 1). Body weight showed a high correlation with all the variables, exceeding 0.70.

**Figure 3:**
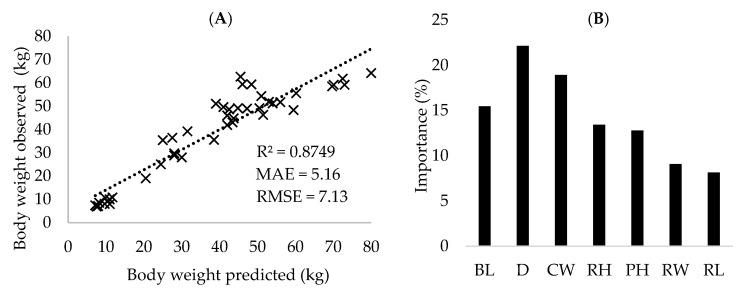
(**A**) Regression of observed weight (body weight) on predicted weight in dairy goats and (**B**) percentage of importance of variables extracted from digital images for body weight prediction. R^2^: coefficient of determination; MAE: mean absolute error; RMSE: root mean square error; BL: body length; D: chest depth; CW: chest width; RH: rump height; PH: paw height; RW: rump width; RL: rump length.

The random forest model was precise (R^2^: 0.8749) and accuracy (MAE: 5.16 and RMSE: 7.13) to predict the body weight using seven morphometric features extracted from digital images of dairy goats (Figure 3A). Among the image-derived variables (Figure 3B), D was the most influential, explaining 22.14% of the variation in body weight prediction, followed by CW (18.93%), BL (15.47%), RH (13.44%), PH (12.79%), RW (9.08%), and RL (8.15%).

### 3.2. Classification of Body Score Condition

**Figure 4:**
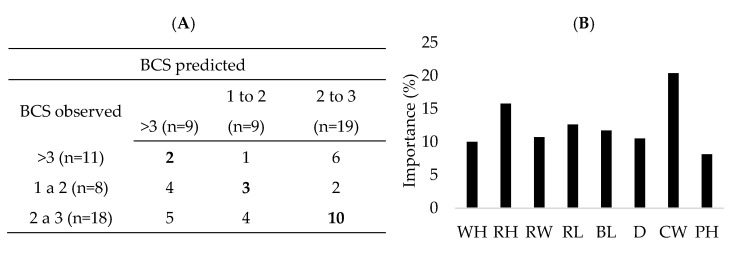
(**A**) Confusion matrix of the test set and (**B**) percentage of importance of variables extracted from digital images for body condition score classification in dairy goats. n = number of observations within each class. BCS: body condition score; WH: withers height; RH: rump height; RW: rump width; RL: rump length; BL: body length; D: chest depth; CW: chest width; PH: paw height.

For BCS classification, the random forest model using eight morphometric features from digital images had an error rate of 0.5945 and an accuracy of 0.4054, with the diagonal of the confusion matrix representing the true positives (Figure 4A). The importance of each variable for BCS classification revealed that chest width (CW) made the greatest contribution (20.38%), followed by RH at 15.78%, RL at 12.63%, BL at 11.73%, RW at 10.74%, D at 10.53%, WH at 10.02%, and PH at 8.15% (Figure 4B).

## 4. Discussion

Positive and significant correlations were observed between all morphometric features extracted from the images and body weight. Among them, body length (BL), chest width (CW), and depth (D) exhibited the highest correlations with body weight (*r* > 0.90). This is since measurements such as height, depth, and chest width are closely related to the animal’s body volume, which is in turn strongly associated with body weight. Similarly, Çakmakçi et al. [11], who evaluated morphometric features for predicting the body weight of Norduz sheep using a random forest model, found that BL, D, and CW were the most strongly correlated variables, with correlations of 0.57, 0.77, and 0.72, respectively. For lactating Saanen goats, Pesmen and Yardimci [12] observed that BL and D showed a high correlation with BW (*r* > 0.70).

In this context, Sabry et al. [13] indicates that linear body measurements have the potential to accurately predict the body weight of Shami goats using linear regression models. In addition, Song et al. [14] demonstrated that morphometric features derived from digital images offer the advantage of predicting body weight, enabling continuous monitoring to assess the growth stage and development of the animals. In this study, the random forest model predicted the body weight of female goats with high precision (R^2^ = 0.87) using morphometric features derived from images. This result falls within the range reported by Pesmen and Yardimci [12], who found R^2^ values between 0.71 and 0.95 for predicting the body weight of dairy goats using morphometric measurements.

Consistent with the correlation results, the most influential variables in the random forest model were D, CW and BL. Çakmakçi et al. [11] also reported that D and CW accounted for a substantial portion of the variance, explaining 38.77% and 23.77%, respectively. Regarding the importance of morphometric traits, BL is an example of predictor of body weight. According to Rahman et al. [15], BL is closely associated with the development of skeletal and muscular systems, which contribute significantly to overall body mass. Moreover, Tyasi et al. [16] observed through simple linear regression that each 1 cm increase in BL corresponds to an approximate increase of 0.58 kg in the body weight of female goats.

Despite the high precision in predicting body weight using image-derived data, the model still exhibited some errors, as indicated by the MAE (5.16) and RMSE (7.13) values. These errors may be attributed to the natural activity level of goats, which can lead to motion-induced distortions in the images and affect the quality of the captured frames. Another contributing factor could be the length and density of the animals’ hair, as image-based measurements may reflect the outer hair surface rather than the actual body contour, potentially biasing the results [5]. Therefore, it is essential to continuously improve image quality and refine the selection of anatomical reference points to enhance prediction accuracy [14].

In the context of BCS prediction, it was revealed that goats with BCS 2 to 3 were better classified, likely due to the larger number of measurements available for this class. However, the overall results indicate that the model has limited ability to accurately classify the condition of these animals due to high error rates and low accuracy. For example, the Alvarez et al. [17] showed that a BCS of 4.5 for Holstein cows could not be classified due to the limited amount of data points available. Tememos et al. [18] reported a similar issue in their study of classifying body condition score (BCS) using digital images of the Greek local breed of dairy goats. They suggested reclassifying BCS as follows: BCS ≤ 2.5 as Thin, 2.5 < BCS ≤ 3.25 as Normal, and BCS > 3.25 as Fat. Their study demonstrated an improvement in prediction accuracy, achieving 0.9794 using a convolutional neural network (CNN) model.

In terms of the importance of each variable in predicting BCS, CW showed the greatest contribution to the model. This can be explained by the fact that, in goats, the greatest fat deposition is found in the sternal region rather than the back regions. These findings corroborate the study by Mendizabal et al. [19] where greater accuracy was observed in estimating the total fat of Blanca Celtibérica female goats using the BCS for the sternal region with an R^2^ of 0.90 compared to the BCS for the lumbar region with an R^2^ of 0.59.

## 5. Conclusions

It was possible to predict the body weight of dairy goats with high precision and accuracy using morphometric features extracted from digital images. However, the precision and accuracy of body condition score classification in female dairy goats were low, indicating that improvements in the image database or the extracted new features are necessary.

## Figures and Tables

**Table 1:** Summary of the database and correlation of BW for the image features evaluated in dairy goats.

Variable	Mean	SD	Min	Max	*r*
Body weight, kg	39.79	20.47	4.90	80.00	
BCS	2.72	0.76	1.00	4.50	
Image measurements (cm)					
Withers Height	78.71	17.09	37.23	101.48	0.86
Rump Height	80.63	17.51	38.20	102.19	0.86
Rump Width	21.40	5.77	7.77	30.93	0.85
Rump Length	25.84	6.84	7.84	40.03	0.72
Body Length	77.69	18.25	35.30	109.39	0.92
Chest Depth	37.67	8.81	17.24	55.05	0.91
Chest Width	23.51	5.18	12.63	34.77	0.92
Paw Height	42.57	9.37	18.46	54.99	0.79

SD: Standard deviation, Min: minimum; Max: maximum; BCS: body condition score; *r*: Pearson correlation with Body Weight.

## Data Availability

The code can be requested from the corresponding authors. The data are not publicly available due to being part of an ongoing study and privacy.

## References

[1] Koyuncu M., Altinçekiç Ş.Ö. (2013). Importance of body condition score in dairy goats. Maced. J. Anim. Sci..

[2] Widiyono I., Sarmin S., Yanuartono Y. (2020). Influence of body condition score on the metabolic and reproductive status of adult female Kacang goats. J. Appl. Anim. Res..

[3] Martins B.M., Mendes A.L.C., Silva L.F., Moreira T.R., Costa J.H.C., Rotta P.P., Chizzotti M.L., Marcondes M.I. (2020). Estimating body weight, body condition score, and type traits in dairy cows using three dimensional cameras and manual body measurements. Livest. Sci..

[4] Ma W., Qi X., Sun Y., Gao R., Ding L., Wang R., Peng C., Zhang J., Wu J., Xu Z. (2024). Computer vision-based measurement techniques for livestock body dimension and weight: A review. Agriculture.

[5] Li K., Teng G. (2022). Study on body size measurement method of goat and cattle under different backgrounds based on deep learning. Electronics.

[6] Zhang A.L., Wu P., Jiang C.X.H., Xuan D.C.Z., Ma E.Y.H., Zhang F.Y.A. (2018). Development and validation of a visual image analysis for monitoring the body size of sheep. J. Appl. Anim. Res..

[7] Iqbal F., Waheed A., Faraz A. (2022). Comparing the predictive ability of machine learning methods in predicting the live body weight of Beetal goats of Pakistan. Pak. J. Zool..

[8] Russel A.J.F., Doney J.M., Gunn R.G. (1969). Subjective assessment of body fat in live sheep. J. Agric. Sci..

[9] ADGA (2022). Guidebook.

[10] Callegari-Jacques S.M. (2003). Bioestatística: Princípios e Aplicações.

[11] Çakmakçi C. (2022). Live weight prediction in Norduz Sheep using machine learning algorithms. Turk. J. Agric.-Food Sci. Technol..

[12] Pesmen G., Yardimci M. (2008). Estimating the live weight using some body measurements in Saanen goats. Arch. Zootech..

[13] Sabry A., Rahman A.E., Shoukry M.M., Mohamed M.I., Salman F.M., Abedo A.A. (2019). Somebody measurements as a management tool for Shami goats raised in subtropical areas in Egypt. Bull. Natl. Res. Cent..

[14] Song X., Bokkers E.A.M., Van der Tol P.P.J., Koerkamp P.G., Van Mourik S. (2018). Automated body weight prediction of dairy cows using 3-dimensional vision. J. Dairy Sci..

[15] Rahman A.S., Khandoker M.A.M.Y., Husain S.S., Apu A.S., Mondal A., Notter D.R. (2008). Morphometric characterization and relationship of body weight with linear body measurements in Black Bengal buck. Bangladesh J. Anim. Sci..

[16] Tyasi T.L., Mathapo M.C., Mokoena K., Maluleke D., Rashijane L.T., Makgowo K.M., Danguru L.W., Molabe K.M., Bopape P.M., Mathye N.D. (2020). Assessment of the relationship between body weight and morphological traits of South African nondescript indigenous goats. J. Anim. Health Prod..

[17] Alvarez J.R., Arroqui M., Mangudo P., Toloza J., Jatip D., Rodríguez J.M., Teyseyre A., Sanz C., Zunino A., Machado C. (2018). Body condition estimation on cows from depth images using Convolutional Neural Networks. Comput. Electron. Agric..

[18] Temenos A., Voulodimos A., Korelidou V., Gelasakis A., Kalogeras D., Doulamis A., Doulamis N. (2024). Goat-CNN: A lightweight convolutional neural network for pose-independent body condition score estimation in goats. J. Agric. Food Res..

[19] Mendizabal J.A., Dela R., Arana A., Purroy A. (2010). A comparison of different pre- and post-slaughter measurements for estimating fat reserves in Spanish Blanca Celtiberica goats. Can. J. Anim. Sci..

